# Evaluating the Causal Effects of Gestational Diabetes Mellitus, Heart Disease, and High Body Mass Index on Maternal Alzheimer’s Disease and Dementia: Multivariable Mendelian Randomization

**DOI:** 10.3389/fgene.2022.833734

**Published:** 2022-06-21

**Authors:** Jie Sheng, Jundong Liu, Kei Hang Katie Chan

**Affiliations:** ^1^ Department of Biomedical Sciences, City University of Hong Kong, Hong Kong, Hong Kong SAR, China; ^2^ Department of Electrical Engineering, City University of Hong Kong, Hong Kong, Hong Kong SAR, China; ^3^ Department of Epidemiology, Centre for Global Cardiometabolic Health, Brown University, Providence, RI, United States

**Keywords:** gestational diabetes mellitus (GDM), heart disease (HD), high body mass index, maternal alzheimer’s disease and dementia, multivariable mendelian randomization (MVMR)

## Abstract

**Introduction:** Gestational diabetes mellitus (GDM), heart disease (HD) and high body mass index (BMI) are strongly related to Alzheimer’s disease (AD) dementia in pregnant women. Therefore, we aimed to determine the total effects of GDM, heart disease, and high BMI on maternal AD dementia.

**Methods:** We used data from the genome-wide association studies of European populations including more than 30,000 participants. We performed two-sample Mendelian randomization (MR) and multivariable MR (MVMR) to systematically estimate the direct effects of GDM, HD, and high BMI on maternal AD and dementia. Multiple sensitivity analyses involving classical MR approaches and expanded MR-pleiotropy residual sum and outlier analysis.

**Results:** In two-sample MR analysis, the inverse-variance weighted method in our study demonstrated no significant causality between GDM and maternal dementia (*β* = −0.006 ± 0.0026, *p* = 0.82). This method also revealed no significant causality between high BMI and maternal dementia (*β* = 0.0024 ± 0.0043, *p* = 0.57), and it was supported by the MR-Egger regression results, which showed no causal effect of high BMI on maternal Alzheimer’s disease and dementia (*β* = 0.0027 ± 0.0096, *p* = 0.78). The IVW method showed no significant causal relationship between maternal HD and maternal Alzheimer’s disease and dementia (*β* = −0.05 ± 0.0042, *p* = 0.117) and MR-Egger regression analysis gave a similar result (*β* = −0.12 ± 0.0060, *p* = 0.079). In MVMR analysis, we found no significant causal relationship between GDM, high BMI, or HD and maternal Alzheimer’s disease and dementia (*p* = 0.94, 0.82, and 0.13, respectively). Thus, the MVMR estimates were consistent with our results from the two-sample MR analysis. We confirmed that these results showed no horizontal pleiotropy and enhanced the robustness of our results through multiple sensitivity analyses.

**Conclusion:** In two-sample MR analysis, we found no significant causal relationship between GDM, HD, high BMI and maternal AD and dementia. These results differed from previous observational studies showing HD is a significant predictor of dementia. MVMR analysis supported no significant causal relationship between GDM, HD, high BMI and maternal AD and dementia. Sensitivity analysis broadly increased the robustness of two-sample MR and MVMR analysis results.

## Introduction

Gestational diabetes mellitus (GDM) is a heterogeneous disease with intricate factors both from genetic and environmental risks. GDM is prone to double from 2016 to 2030 (Koivusalo et al., 2016) globally. GDM generally occurs in pregnant women diagnosed with insulin resistance, and GDM women are more likely to develop type 2 diabetes (T2D) after delivery (Vounzoulaki et al., 2020). Instead of T2D, GDM with obesity not only posed a significant threat to pregnant women, but developed a deteriorated risk to the next generation, which is urgent to establish a comprehensive investigation for GDM prevention (Koivusalo et al., 2016). Current studies found complex associations between GDM, HD, high BMI and AD/dementia (Deckers et al., 2017; Vacínová et al., 2017; Anjum et al., 2018). For instance, population-based cohort studies reported that GDM might induce dementia, and conducted a follow-up survey among 550 GDM women with T2D and AD. Through extracting the genetic variants of GDM, T2D and AD by genotyping analysis, one gene, *PICALM* (rs3851179) related to AD, was significantly associated with a higher risk of GDM (Vounzoulaki et al., 2020). Observational studies have further reported that GDM is associated with cognitive dysfunction and dementia (Keskin et al., 2015). In detail, the Montreal Cognitive Assessment (MoCA) and Symbol Digit Modalities Test (SDMT) scores have been shown to be significantly lower in GDM patients than in normal subjects, indicating that (*p* = 0.003 and 0.004, respectively) (Keskin et al., 2015). Similarly, obesity is usually associated with a high body mass index (BMI), and BMI greater than 30 has been shown to exacerbate GDM (*p* < 0.001). Moreover, an increase in adjusted BMI by one standard deviation due to genetic factors increased the risk of HD (odds ratio: 1.46, 95% confidence interval: 1.32, 1.02) (Emdin et al., 2017). From another perspective, a longitudinal cohort study in 2021 showed that HD and HD-related factors might alter the risk of dementia (Grande et al., 2020). Furthermore, a high BMI (≥30 kg/m^2^) combined with metabolic syndrome increased the risk of GDM in pregnant women.

Observational studies have reported multiple associations between GDM, HD, obesity and AD/dementia, but these studies are susceptible to potential confounding effects. The associations did not stand for the causality between GDM, HD, high BMI and dementia, moreover, the causality demonstrated in observational studies might lack statistical validity due to confounding factors such as inconsistent designs, conflicting results and high heterogeneity in the settings of observational studies. The meta-analysis in 2017 (Deckers et al., 2017) emphasized the correlations between coronary heart disease, and cognitive disorders. However, significant heterogeneity and inconsistent results were found in this analysis. The meta-analysis included 11 cohort studies, one study showed heart disease might significantly induce vascular dementia. But other four cross-sectional studies found no significant evidence that HD could increase the high risk of dementia. High heterogeneity was detected in these statistical results, making the results controversial among these observational studies. Observational findings provided enriching evidence towards the associations between HD, high BMI and AD/dementia, but still could not overcome the influence of confounding factors, meaning that unknown or artificial intervention rooted in observational studies may lead to biased estimation towards the relationship or the causality between exposure(s) and outcome. The latest study published in 2021 (Sana et al., 2021) used statistical analysis to further assess the association between GDM and Alzheimer’s disease from pathological mechanisms and Montreal Cognitive Assessment (MoCA). In this study, Alzheimer’s disease is the most common cause of dementia (Vacínová et al., 2017), and MoCA assessment could effectively evaluate the level of cognitive decline in GDM women. This study investigated 80 GDM women and used statistical analysis to decipher the association between GDM and Alzheimer’s disease. The MoCA’s result displayed a significant decline compared with other control groups in terms of attention, executive, memory and visuospatial function (P<0.05) . From the perspective of pathological mechanisms, the study also found the serum dipeptidyl peptidase-4 (DPP4) related to GDM also could significantly alter the risk of dementia in pregnant women (P<0.05) . What we need to dig further from this study is that, besides expanding the sample size, considering GDM involves other complex complications, not limited to risk factors of DPP4, how to systematically further investigate the relationship between GDM with other related complications that is the central point in our study.

To solve the problems mentioned above, we decided to use Mendelian randomization (MR) to overcome the biased estimation and reverse causation towards the relationship between GDM, HD, high BMI and AD/dementia due to confounding factors. Besides, MR analysis combines bioinformatics, statistics with genetics, using genetic variants [single nucleotide polymorphisms (SNPs)] as the instrumental variables to estimate whether a causal relationship existed between exposure(s) and outcome by statistical methods. Genetic variants are almost impossible to be influenced by confounders because they are distributed randomly at conception throughout life, indicating that if genetic variants are randomly distributed in the population, the causality between exposure(s) and outcome will be due to genetic inheritance, not other confounding factors such as environmental risks, lifestyle, social status and so on (Bowden et al., 2015). Simultaneously, MR analysis introduces the concept of instrumental variables (IVs) from statistics, through applying the classical least-squares principle, to achieve the unbiased estimation towards the causal relationship between exposure(s) and outcome, and eliminate the influence from confounding factors. Generally, MR analysis is based on three essential assumptions: 1) The genetic variants are instrumental variables (IVs) highly related to the exposure; 2) IVs are not related to confounders (Katan, 2004); and 3) the genetic variants have a specific effect on the targeted outcome, but only *via* independent exposure factors and not *via* other factors (Bowden et al., 2015). Besides, MR has a conceptual resemblance to randomized controlled trials (RCTs), with randomization occurring at meiosis. When RCTs are impractical or unethical, MR analysis can effectively overcome these intractable problems in RCT (George D. Smith and Ebrahim, 2003). Recently, databases from genome-wide association studies (GWASs) have provided flexible, strong support for MR analysis to identify causal relationships between specific exposure factors and outcomes.

Multivariable MR (MVMR) is an advanced approach that allows for the simultaneous evaluation of the effects of separate, but potentially relevant exposure factors on specific outcomes (Katan, 2004). In MVMR, genetic variants are permitted to be relevant to more than one reliable exposure factor, provided that they are not associated with confounding factors that exist in any of the exposure–outcome relationships and do not affect those outcomes directly. The IV assumptions in MVMR are similar to those in the two-sample MR analysis, except that a series of IVs are permitted.

This study had two aims. First, we used a two-sample MR design to investigate the causal effect of each exposure factor on maternal dementia, using publicly available large GWAS databases with data on genetic variants related to each exposure factor and outcome. Second, we used MVMR analysis to disentangle the overall causal relationship between exposure factors (GDM, HD, and high BMI) and the outcome (maternal AD/dementia). The total and direct causal effects of GDM, HD, and high BMI on AD/dementia were further delineated in this study.

## Materials and Methods

**FIGURE 1:**
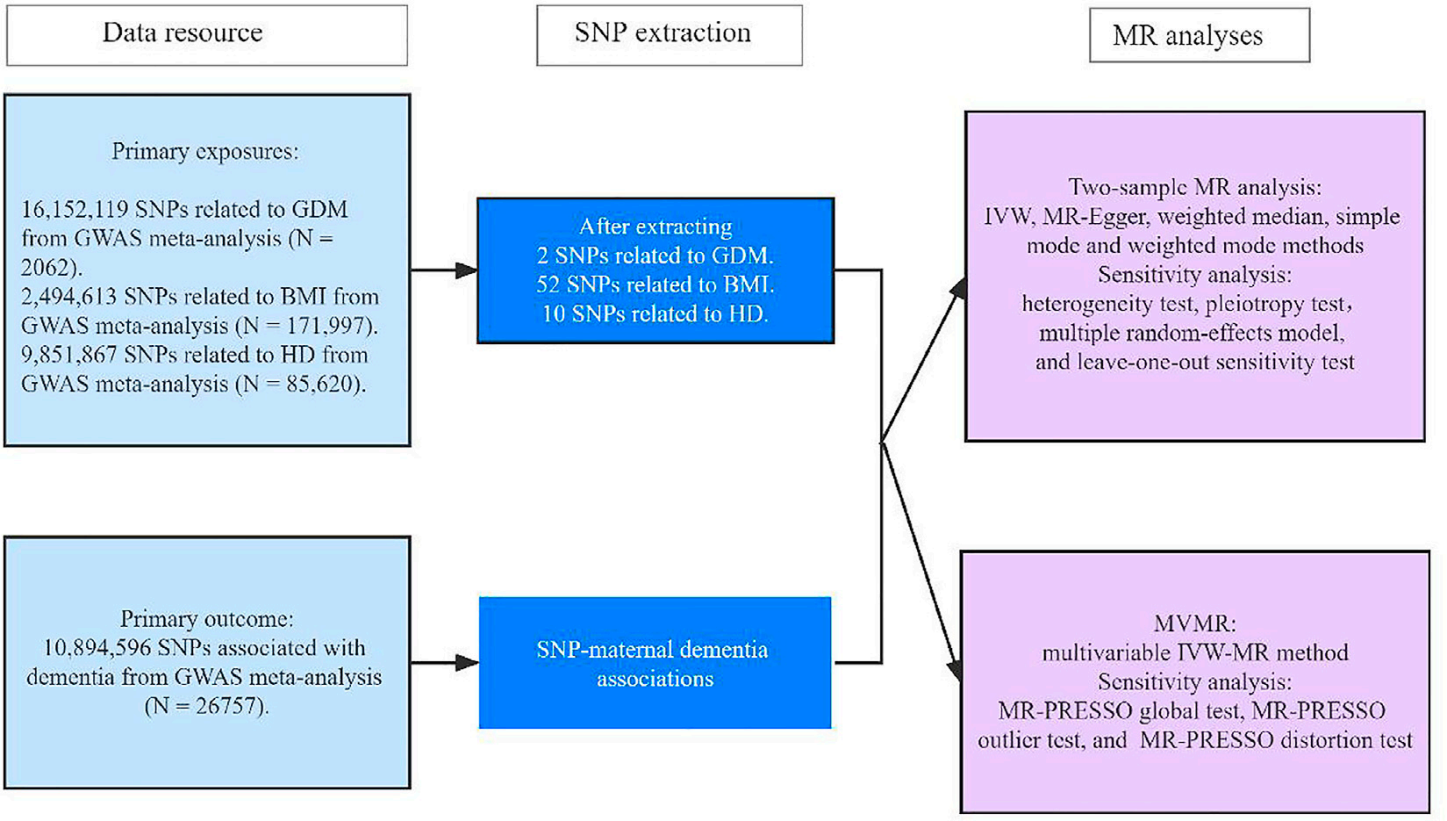
Research framework. GDM, gestational diabetes mellitus; BMI, body mass index; HD, heart disease; GWAS, genome-wide association study; IVW, inverse-variance weighted; MR-PRESSO, pleiotropy residual sum and outlier.

We used two-sample MR and MVMR analyses to determine the independent and total causal effects of GDM, high BMI, and HD on dementia endpoints in European female populations from a GWAS summary database (Figure 1). All participants in studies included in the review provided informed consent to participate in the original studies. Specific ethical approval was not required for this study, as it reported summary-level data.

### GDM-Related Instrumental Variables

GDM data was predominantly extracted from GWAS summary data from the European populations in 2020. The data included 16,152,119 SNPs related to GDM and 2,062 identified GDM cases were used in our MR analysis (https://storage.googleapis.com/finngen-public-data-r2/summary_stats/finngen_r2_GEST_DIABETES.gz). In the Finnish Gestational Diabetes Study, GDM was defined as the GDM status at 75 g OGTT during 12–16 weeks, or at 24–28 weeks of gestation stage. Fasting plasma glucose ≥5.3 mmol/L, 1-hour glucose ≥10.0 mmol/L or 2-hour glucose ≥8.6 mmol/L (Keikkala et al., 2020). The basic criteria for IVs that were significantly associated with exposure factors were: 1) Meeting the genome-wide significant threshold (*P* = 5e^−8^) (Lawlor et al., 2008) and 2) no linkage disequilibrium (LD) with other SNPs to ensure that we obtained genetically independent SNPs (Lawlor et al., 2008). In our study, we used the “TwoSampleMR” package of R 4.1.0 version to extract IVs that were significantly related to GDM, HD and high BMI and prune SNPs with potential LD among exposures *via* a moderate screen criterion ( $r^{2}=0.05,kb=1000),\;$ the scale of $r^{2}$ is 0∼1, $r^{2}=1$ means SNPs with entire LD, thus the smaller $r^{2}$ indicates the less likely to occur LD among SNPs. kb represents the region where SNPs appear LD at their genetic loci. Generally, the scale of kb is 500∼50,000, the more distant among SNPs, the less likely to occur LD among SNPs (Hemani et al., 2018). After data extraction, two SNPs that met these requirements were included in the subsequent harmonizing step to perform two-sample MR and MVMR analyses.

### BMI-Related Instrumental Variables

We identified SNPs strongly related (*p* < 5e^−8^) to a high BMI ( $BMI\geq 30kg/m^{2}$ ) from GWAS data of European ancestry that were published in 2015 (Locke et al., 2015) and screened these SNPs using a moderate linkage disequilibrium (LD) criterion ( $r^{2}=0.05,kb=1000$ ). For data from this database, a two-stage meta-analysis was used to identify BMI-related loci in the European population. In stage one, 234,069 subjects from 80 GWASs of BMI were used, and an additional 34 BMI-related findings involving 88,137 subjects were accepted. These subjects were used for secondary meta-analyses and divided into: 1) The total European population, 2) European men, 3) European women, and 4) subjects of all ancestries. This database was supported by the Genetic Investigation Anthropometric Traits (GIANT) consortium. After reviewing 2,494,613 BMI-related SNPs, 171,997 female cases were identified and eventually included in our MR analysis. After extracting the data, 52 SNPs met the requirements and were incorporated into the subsequent harmonizing procedure to complete the two-sample MR analysis, and 31 targeted SNPs were used for the MVMR analysis.

### HD-Related Instrumental Variables

We identified SNPs from a large-scale GWAS meta-analysis of the European population in 2018 (https://gwas.mrcieu.ac.uk/datasets/ukb-b-12477/) that were closely associated with HD (*p* < 5e^−8^) and screened them *via* a moderate LD criterion ( $r^{2}=0.05,kb=1000$ ). This database was supported by the United Kingdom biobank study, participants in this study were enquired about “How did your mother ever suffer from heart disease,” and heart disease was classified as one of the chronic diseases investigated in the United Kingdom Biobank (Trehearne, 2016). This database collected 9,851,867 HD-related SNPs and 85,620 female HD cases, and we used 85,620 female HD cases for MR analyses. After extracting the data, ten SNPs that met the requirements were included in the subsequent harmonizing step to implement the two-sample MR and MVMR analyses.

### Genetic Information Related to Maternal Alzheimer’s Disease/Dementia

Maternal Alzheimer’s disease (AD)/dementia data was derived from a 2017 GWAS summary database of the European population supported by the United Kingdom biobank (https://gwas.mrcieu.ac.uk/datasets/ukb-a-210/). This database comprised data on 10,894,596 dementia-associated SNPs from 26,757 maternal AD dementia cases. In this data, the AD/dementia was mainly defined as the AD dementia syndrome, which belongs to the anamnestic syndrome of hippocampal type, with the relevant decline in memory, executive function, attention, word-finding, spatial cognition (Karantzoulis and Galvin, 2011); (Klinedinst et al., 2020). Besides, the United Kingdom biobank study reported the genetic risk factor rooted in AD dementia, such as apolipoprotein E ε4 allele (APOE4), and a parental family history of AD dementia. We used these 26,757 maternal AD dementia cases for harmonizing SNPs related to GDM, HD, and high BMI to perform the two-sample MR and MVMR analyses. All databases used for the two-sample MR and MVMR analyses are included in Supplementary Datas S1–S3.

### Statistical Approaches and Sensitivity Analyses

First, we used two-sample MR analysis to assess the significance level of the causal effect of each exposure factor on maternal dementia. We mainly performed inverse-variance weighted (IVW) meta-analysis and MR-Egger regression analysis, along with weighted median, simple mode, and weighted mode approaches, to determine whether existed a causal effect of each exposure factor on maternal dementia (Hemani et al., 2018); (Bowden et al., 2015); (Bowden et al., 2016). IVW is an integral approach used to realize unbiased estimations, and it ensures the appropriate statistical power to detect a causal relationship between an exposure factor and an outcome. MR-Egger regression is similar to IVW, except it includes an intercept term for the average pleiotropic effect. The intercept in the MR-Egger regression can be used to test the IV hypothesis. When an average pleiotropic effect exists, the intercept is not zero, indicating that the IV assumptions are invalid. As a type of sensitivity analysis, the heterogeneity test was primarily used to detect significant outliers in each IV and thus guarantee the validity of the IV assumptions. We used IVW, multiple random-effects modeling, and the MR-Egger intercept to perform a heterogeneity test. We then used the pleiotropy test (mainly the MR-Egger intercept) to identify significant outliers in multiple IVs. Our sensitivity analysis also included a “leave-one-out” sensitivity test to monitor significant differences in our models before and after excluding each IV (Zheng et al., 2017). Scatter plots and funnel plots were also used to visualize and detect outliers to draw consistent conclusions with the sensitivity analysis described above.

Second, we used MVMR analysis to perform a multivariable linear regression, combining the exposure factors (GDM, high BMI, and HD) with maternal dementia. We mainly used the multivariable IVW method for MVMR analysis (Katan, 2004) to calculate the causal effects of GDM, high BMI, and HD on maternal dementia. To enhance the robustness of the model, we performed a sensitivity analysis using the MR-pleiotropy residual sum and outlier (MR-PRESSO) method (Verbanck et al., 2018), and we used a global test to detect horizontal pleiotropy in our multivariable linear regression model. After correcting for pleiotropy, we used an outlier test to detect outliers. Moreover, we used a distortion test to determine significant differences before and after correcting pleiotropy.

Additional sensitivity analyses were necessary to enhance the robustness of two-sample MR and MVMR models. In the two-sample MR analysis, a heterogeneity test, a pleiotropy test, and a “leave-one-out” sensitivity test were used to detect heterogeneity, rectify horizontal pleiotropy, and remove outlier variants, respectively. In the MVMR model, MR-PRESSO global tests were mainly used to detect potential outliers, correct for horizontal pleiotropy, and resolve heterogeneity. All analyses were performed using R software version 4.0.4, with “two-sample MR” R package version 3.5.3 and MR-PRESSO version 1.0 (https://www.r-project.org/).

## Results

### Results of Two-Sample MR Analysis of Exposure Factors and Maternal Dementia

Table 1 show the results of two-sample MR analysis regarding the effects of exposures on maternal AD/dementia, as determined through multiple methods. First, we identified two SNPs strongly associated with GDM (*p* = 5e^−8^, Supplementary Data S1), and the maternal AD/dementia-related information was independently extracted ( $r^{2}=0.05,\;kb=1000$ , Supplementary Data S2). By harmonizing these SNPs, we could calculate their causal effects on GDM and maternal dementia. As shown in Table 1, there was no causal relationship between GDM and maternal dementia using IVW method (*β* = −0.0006 ± 0.0026, *p* = 0.82). We then analyzed 52 SNPs strongly associated with high BMI (*p* = 5e^−8^, Supplementary Data S1), and those SNPs were independently extracted ( $r^{2}=0.05,\;kb=1000$ ) from genetic information related to maternal AD/dementia (Supplementary Data S2). By harmonizing these SNPs, we could detect the causal effect of high BMI on maternal dementia. As shown in Table 1, no significant causal relationship between high BMI and maternal dementia was identified using the IVW method (*β* = 0.0024 ± 0.0043, *p* = 0.57). The MR-Egger method also showed no support for a causal relationship between high BMI and maternal dementia (*β* = 0.0027 ± 0.0096, *p* = 0.78). The effect direction was consistent across all MR methods (weighted median, simple mode, and weighted mode).

**TABLE 1:** | Two-sample MR result: the causal effect of GDM, high BMI, HD on maternal AD/dementia.

Exposure	Method	N(SNPs)	*β*	Standard deviation	p-Value	Outcome
GDM	Inverse-variance weighted	2	−0.0006	0.0026	0.82	Alzheimer’s disease and Dementia
High BMI	Inverse-variance weighted	52	0.0024	0.0043	0.57	Alzheimer’s disease and Dementia
High BMI	MR-Egger intercept	52	0.0027	0.0096	0.78	Alzheimer’s disease and Dementia
High BMI	Simple mode	52	0.000068	0.0033	0.98	Alzheimer’s disease and Dementia
High BMI	Simple mode	52	0.0065	0.0092	0.49	Alzheimer’s disease and Dementia
High BMI	Weighted mode	52	0.0033	0.0070	0.64	Alzheimer’s disease and Dementia
HD	Inverse-variance weighted	10	−0.05	0.0042	0.117	Alzheimer’s disease and Dementia
HD	MR-Egger intercept	10	−0.12	0.0060	0.079	Alzheimer’s disease and Dementia
HD	Simple mode	10	0.08	0.0035	0.157	Alzheimer’s disease and Dementia
HD	Simple mode	10	0.08	0.0090	0.423	Alzheimer’s disease and Dementia
HD	Weighted mode	10	−0.10	0.0050	0.063	Alzheimer’s disease and Dementia

N (SNPs) stands for the number of SNPs in GDM, high BMI and HD. *β* stands for the estimated coefficient calculated by multiple methods, including inverse-variance weighted, MR-Egger intercept, simple mode, weighted mode methods in our two-sample MR analysis.

Additionally, we identified 10 SNPs strongly associated with HD (*P* = 5e^−8^, Supplementary Data S1), and those SNPs were independently extracted $\left(r^{2}=0.05,kb=1000\right)$ from genetic information related to maternal dementia (Supplementary Data S2). By harmonizing the data on these SNPs, we further evaluated the causal relationship between HD and maternal dementia. As shown in Table 1, there was no significant causal relationship between maternal HD and dementia using the IVW approach (*β* = −0.05 ± 0.0042, *p* = 0.117). Similarly, the MR-Egger method did not support the causal relationship between BMI and maternal dementia (*β* = −0.12 ± 0.0060, *p* = 0.079). The effect direction was consistent across all MR methods (simple mode and weighted mode).

### Sensitivity Analyses of the Effects of Exposure Factors on Maternal Dementia

The results of sensitivity analyses regarding the effects of exposure factors on dementia are shown in Supplementary Tables S2–S7 in Supplementary Data S3. As shown in Supplementary Table S2, there was no significant heterogeneity between GDM and maternal dementia (*p* = 0.11). However, the data presented in Supplementary Table S3 show significant heterogeneity between BMI and maternal dementia using the IVW (*p* = 0.034) and MR-Egger (*p* = 0.028) methods. Therefore, we corrected heterogeneity using multiple random-effects modeling (Supplementary Table S4). As shown in Supplementary Table S5, there was no significant pleiotropy between BMI and maternal dementia *via* an MR-intercept test (*p* = 0.77). The data presented in Supplementary Table S6 demonstrate no significant heterogeneity between HD and maternal dementia (*p* = 0.15), and those presented in Supplementary Table S7 show no significant pleiotropy between HD and maternal dementia using the MR-intercept test (*p* = 0.17).

**FIGURE 2:**
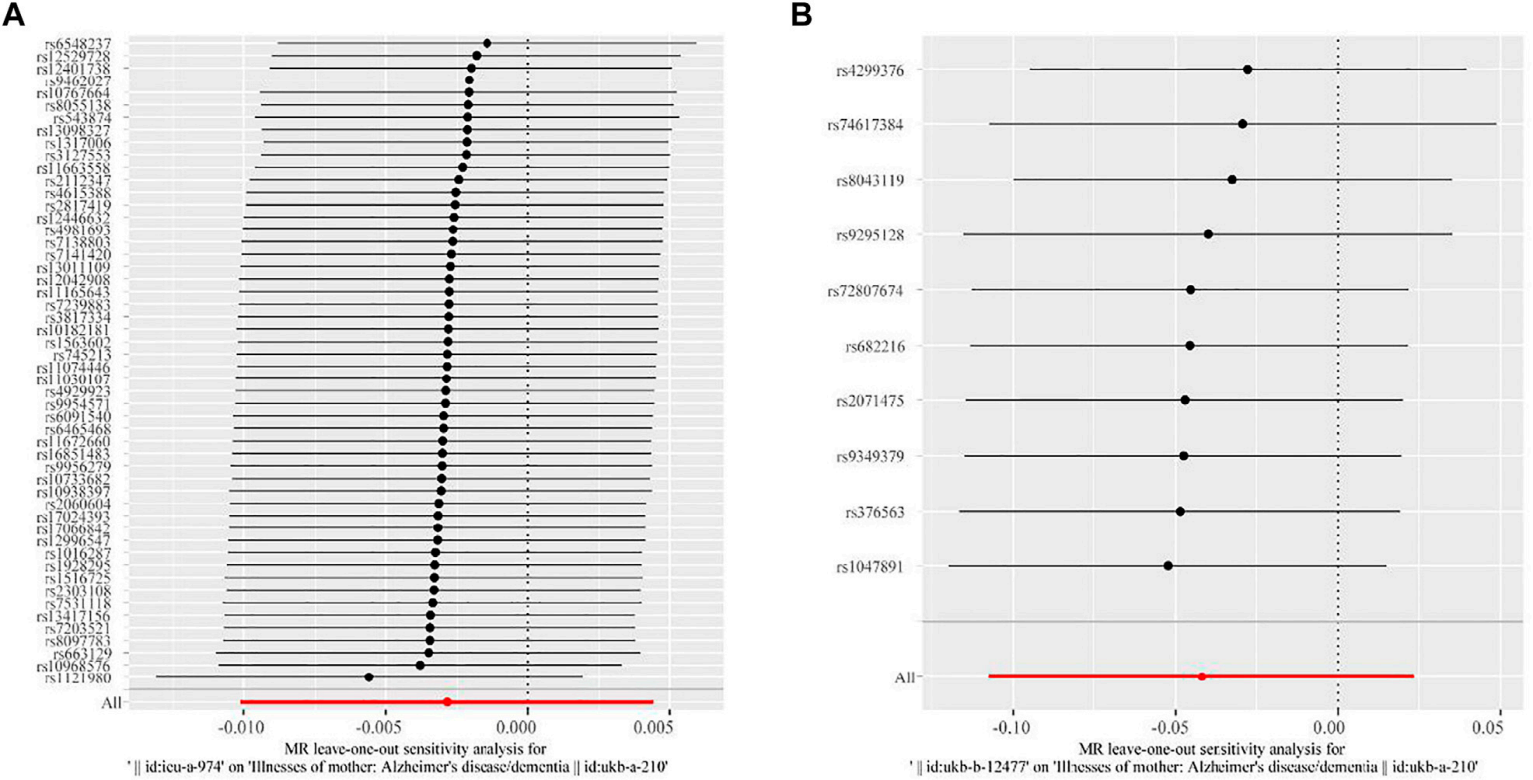
Results of “leave-one-out” sensitivity analysis of the association between high BMI, HD, and maternal dementia in two-sample MR analysis. **(A)** Results for the association between high BMI and maternal dementia. **(B)** Results for the association between HD and maternal dementia. Each black line represents the MR effect after eliminating each SNP at a 95% confidence interval. Each black point represents the median value (that is, the *β* value) across each black line. The red line at the bottom represents the total MR effect in the sensitivity analysis.

Furthermore, we performed “leave-one-out” sensitivity analysis to test the robustness of our MR results related to high BMI, HD, and maternal dementia. The “leave-one-out” sensitivity analysis could not be applied to GDM and maternal dementia due to insufficient SNPs. As shown in Figures 2A,B, each red line is to the left of zero, and each black point in the middle of each black line is to the left of zero, indicating that our fitted results are robust, with no outliers, and thus will not be influenced by the removal of each SNP.

### Visualization of the Exposure Factors and Maternal Dementia

**FIGURE 3:**
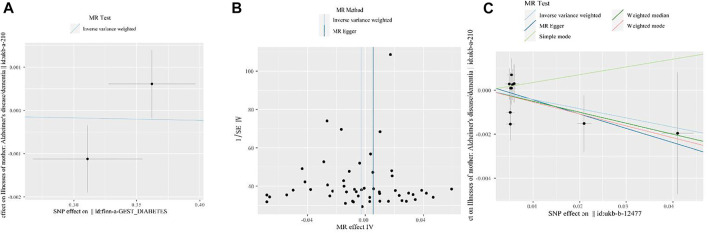
Visualization of the MR analysis results for GDM, high BMI, HD, and maternal dementia. **(A)** Scatter plot for MR analyses of the causal association between GDM and maternal dementia mainly *via* IVW method. The slope of the blue line in the middle indicates the MR effect using the IVW estimation. **(B)** Funnel plot of the causal association between high BMI and maternal dementia. We performed analyses by the classical IVW and MR-Egger tests. The two vertical lines (from left to right) in the middle represent the MR effect using IVW method and MR Egger methods, respectively. **(C)** Scatter plot of the MR effect of HD on maternal dementia determined using multiple methods. Analyses include the IVW and MR-Egger methods, the simple mode, and the weighted median and weighted mode. The slope of each line in the middle indicates the MR effect for each method.

Figure 3 shows the visualization results for GDM, high BMI, HD, and maternal dementia. First, we visualized the causal link between GDM and maternal dementia. In Figure 3A, the blue line in the middle is flat, indicating no MR effect of GDM on maternal dementia. Figure 3B shows a funnel plot to visualize the MR effect of BMI on maternal dementia, as determined by IVW and MR-Egger regression analyses. All SNPs (black points) were distributed symmetrically and evenly on both sides of the central axis (IVW and MR-Egger regression estimation). No outliers deviated significantly from the central axis, indicating that our fitted effect was robust. Figure 3C shows a scatter plot to visualize the MR effect of HD on maternal dementia, assessed using multiple methods. The slope of each line in the middle indicates the MR effect per method. The MR effect of simple mode method was positive, while the other four approaches were negative.

### Results of MVMR and MRPRESSO Sensitivity Test for the Effects of Exposure Factors on Maternal Dementia

In MVMR analysis, we mainly used the multivariable IVW method to calculate the total causal effects of GDM, high BMI, and HD on maternal dementia. Table 2 shows the overall MVMR result using the multivariable IVW method, which showed no significant causal effects of GDM (*p* = 0.94), high BMI (*p* = 0.82), or HD (*p* = 0.13) on maternal AD/dementia.

**TABLE 2:** | Results of MVMR analysis regarding the overall causality between GDM, high BMI, HD and maternal AD/dementia.

Exposure	Method	N (SNPs)	*β*	Standard deviation	p-Value	Outcome
GDM	Inverse-variance weighted	2	−0.0001	0.0018	0.94	Alzheimer’s disease and Dementia
High BMI	Inverse-variance weighted	31	−0.001	0.0046	0.83	Alzheimer’s disease and Dementia
HD	Inverse-variance weighted	5	−0.078	0.051	0.13	Alzheimer’s disease and Dementia

N (SNPs) stands for the number of SNPs in GDM, high BMI and HD. *β* stands for the estimated coefficient calculated by inverse-variance weighted in our MVMR analysis.

We then performed an MVMR sensitivity test based on MR-PRESSO analysis as shown in Supplementary Tables S9–S11 in Supplementary Data S3. Supplementary Table S9 shows the significant pleiotropy revealed by our multivariable linear regression modeling (*p* = 0.004). Hence, we used the MR-PRESSO outlier test to eliminate horizontal pleiotropy and determine whether outliers still existed. As shown in Supplementary Table S10, after eliminating horizontal pleiotropy, no significant outliers existed in the MVMR effect of each exposure on maternal dementia (*p* = 0.43, 0.53, and 0.10 for GDM, high BMI, and HD, respectively) . To confirm the robustness of our multivariable linear regression model, we also used the MR-PRESSO distortion test to detect significant differences across the causal estimations of our MVMR analysis before and after outlier removal. Finally, as shown in Supplementary Table S11, there were no significant differences in our MVMR results before and after eliminating outliers, further indicating the overall robustness of our MVMR results for the causality between GDM, high BMI, and HD and maternal dementia. All tables are presented in the Supplementary Material, and all data used for MVMR analysis are presented in Supplementary Data S4.

## Discussion

The aim of this study was to systematically estimate the gender-specific causal effects of GDM, HD, and high BMI on maternal dementia from large-scale GWASs databases of female European populations. Our MR design allowed us to disentangle the independent and total effects of genetically predicted GDM, HD, and high BMI on maternal dementia. GDM, HD, and high BMI due to genetic variants were not causally related to the risk of maternal dementia when using two-sample MR analysis, whose databases from the GIANT and MRC-IEU (included 259,679 female participants). These non-significant findings were consistent among various sensitivity analyses (IVW, MR-intercept test, multiple random-effects modeling, and “leave-one-out” sensitivity test) used to assess heterogeneity, pleiotropy, and no-bias estimations. Additionally, we found no significant total causal effect of GDM, HD, high BMI on maternal dementia in multivariable Mendelian randomization. The results of the sensitivity analysis (MR-PRESSO test) were broadly consistent with the MVMR results, which also supported our causal inference.

A previous study investigated the association between GDM and dementia, but it lacked sufficient evidence and multiple sensitivity tests to confirm the validity of these results (Vacínová et al., 2017). Vacínová et al. investigated 550 GDM women, Alzheimer’s disease (AD), and type 2 diabetes mellitus (T2D) over a period of 20 years. Genetic variants related to AD and T2D were included in the statistical analysis (mainly chi-square tests) to assess the relationship between GDM and AD. There were no statistically significant associations between candidate genes related to AD (rs381361 in *CR1*, rs744373 in *BIN1*, rs11136000 in *CLU*) and GDM, but one gene, *PICALM* (rs3851179), was significantly associated with a high risk of GDM. Moreover, the study of Vacínová et al. only used chi-square tests with small sample sizes to perform statistical analyses related to GDM and AD, and no sensitivity analysis was performed. In our study, we expanded the sample size of GDM- (*n* = 2062) and dementia-related (*n* = 26,757) database from the GWASs database, which will provide solid data support for our MR analysis results compared to previous studies.

Moreover, we used two-sample Mendelian randomization and MVMR analysis, including sensitivity analyses, to systematically assess the causal effects of GDM, HD, and high BMI on maternal dementia. Our sensitivity analysis results were all consistent with the results obtained by MR. We also found one previous MR study that showed no causal relationship between T2D and dementia in a European population (Thomassen et al., 2020). As T2D and GDM have a similar pathogenesis (Vounzoulaki et al., 2020), this finding supports our MR result regarding GDM and dementia.

A previous systematic review and meta-analysis published in 2017 (Deckers et al., 2017) reported associations between HD, coronary heart disease, and cognitive dysfunction, but there were inconsistent results and a high level of heterogeneity among the studies included in the analysis. In the 11 prospective cohort studies included in that meta-analysis, one study concluded that HD has a significant role in the development of vascular dementia. Four cross-sectional studies that included 623,588 participants found no significant evidence of a relationship between HD and dementia. However, in the meta-analysis that included 10 studies involving 24,801 participants, HD was found to significantly increase the risk of developing dementia. These studies were large-scale population-based investigations with multiple study designs, but major conclusions were drawn from these studies. Moreover, a high level of heterogeneity was detected due to inconsistent methods among the cross-sectional studies. Inconsistent results for the relationship between HD and dementia have also been reported due to different study designs in cross-sectional studies (Anjum et al., 2018). We used MR analysis to ensure consistent results regarding the causal relationship between HD and dementia. In two-sample MR analysis, we found no significant causal effect of HD on maternal dementia using the IVW method and MR-Egger intercept test. This was consistent with the results of our two-sample MR analysis.

A literature review published in 2018 (Anjum et al., 2018), including 15 prospective studies, found that obesity is associated with a higher risk of dementia. In obese individuals, long-term food overconsumption may significantly influence cerebral glucose metabolism and exacerbate the potential risk of developing dementia. However, these observational studies did not account for reverse causation and confounders, leading to potentially inaccurate correlations between BMI and dementia.

For instance, epidemiological studies have found that elevated BMI later in life has a lower impact on dementia. However, MR analysis as the core methodology in current study effectively overcomes the influence of reverse causation and confounders. Moreover, our two-sample MR analysis showed no significant causal effect of maternal HD on dementia. The lack of a causal relationship between GDM and maternal dementia in MVMR analysis was consistent with our two-sample MR analysis results. A previous MR study reporting no significant relationship between BMI and dementia in a European population also supports our results (Nordestgaard et al., 2017).

Our study has three main strengths. First, using data from previous studies, we expanded the sample size of large-scale GWASs of GDM and dementia and systematically studied the causal effects of GDM-related exposure factors (HD, high BMI) on maternal dementia. Second, to the best of our knowledge, there was no measurement bias in our gender-specific MR analysis. After detecting potential pleiotropy between BMI and maternal dementia in two-sample MR analysis, we used multiple random-effects modeling to correct for pleiotropy and ensure the robustness of our model. Similarly, when assessing pleiotropy in MVMR analysis, we used the MR-PRESSO outlier test, effectively guaranteeing the validity of IV assumptions in MR modeling. Thirdly, we also found consistent results both in two-sample MR and MVMR analyses. The non-significant findings in MVMR estimates of GDM, high BMI, HD, and maternal dementia were consistent with the magnitude and direction of the causal genetic effect seen in two-sample MR analysis, broadly confirming the credibility of our conclusion.

Our study also has some limitations that should be noted. First, we focused on clinical diagnoses of maternal dementia rather than neuropathological diagnoses, and our MR results may have been influenced by different diagnoses. Discrepancies between clinical and neuropathological diagnoses of dementia have been reported (Sutherland et al., 2017). Another previous study found that vascular dementia and AD may exacerbate vascular damage, diabetes, and hypertension, based on clinical diagnoses and pathological findings (Arvanitakis et al., 2006). We found that T2D was not causally linked to AD, which is consistent with studies involving neuropathological findings (Abner et al., 2016). Therefore, clinical diagnoses could reasonably be used in our study. Given the similar pathogenic mechanisms of T2D and GDM (Vounzoulaki et al., 2020), these clinical and neuropathological findings support our MR results concerning maternal AD/dementia and GDM. Second, we need to further collect more information regarding how HD was diagnosed in the United Kingdom Biobank study. Third, we acknowledged the lack of power in the GWAS database of GDM. Compared with the current GWAS database of GDM (Pervjakova et al., 2022), the GDM database we collected only included 2062 GDM women, which might influence the statistical power of our MR results as we just extracted only two independent SNPs of GDM. We hope to include more GWAS studies with this complications in the future. Fourth, we will adopt advanced methods such as non-linear Mendelian randomization (Zhou et al., 2021), limited information maximum likelihood (Davies et al., 2015) methods to ensure the validity of SNPs selection in MR analysis. Although genetic variants are likely to be regarded as valid IVs due to their genetic advantages in MR analysis, Mendel’s second law is not applicable for all diseases (Smith and Ebrahim, 2003), especially in those diseases with intricate factors. For instance, in our study, GDM and AD dementia are both with a wide range of risk factors (including environmental risks and genetic inheritance), so besides traditional method of selecting SNPs, it is necessary for us to adopt advanced techniques to ensure the validity of SNPs selection regarding GDM and AD dementia in the future.

## Conclusion

We used MR to evaluate the causal relationship between GDM, maternal HD, or high BMI and maternal dementia. In the two-sample MR analysis, we found no significant causal relationship between GDM, HD, or high BMI and maternal AD/dementia, which differs from the results of previous observational studies showing that HD is a significant predictor of dementia. This discrepancy may be due to confounding factors and reverse causation in previous studies. Our MVMR analysis gave consistent results, revealing no significant causal effect of GDM, HD, or high BMI on maternal AD/dementia. Then we used multiple sensitivity tests to detect potential outliers, correct for horizontal pleiotropy, and resolve heterogeneity, but due to the limited samples of GDM, our robustness of sensitivity tests still needs to be verified in the future. Future investigations of the potential risks of GDM, heart disease, high BMI and other relevant complications in maternal AD/dementia are necessary when larger datasets of these diseases become available.

## Data Availability

The datasets presented in this study can be found in online repositories. The names of the repository/repositories and accession number(s) can be found in the article/Supplementary Material.
