# Supplementary material for: Evaluating the Causal Effects of Gestational Diabetes Mellitus, Heart Disease, and High Body Mass Index on Maternal Alzheimer’s Disease and Dementia: Multivariable Mendelian Randomization
Source: Front Genet. 2022 Jun 21;13:833734. doi: 10.3389/fgene.2022.833734 (PMC9255379; doi:10.3389/fgene.2022.833734)
Supplement: Supplementary file 1 [file DataSheet1.pdf]

Supplementary Data 1: Strong IVs related to exposures by screening LD

Exposure	Chromosome	Position	*β*	Standard deviation	*P*-value	Effect allele	Other effect allele	Sample size	Outcome
GDM	rs61872784	10	0.3112	0.0433	$5e^{-8}$	A	T	2062	Alzheimer's disease and Dementia
GDM	rs10830963	11	0.3621	0.0344	$5e^{-8}$	G	C	2062	Alzheimer's disease and Dementia
High BMI	rs543874	1	-0.0018	0.005	$5e^{-8}$	G	A	171997	Alzheimer's disease and Dementia
High BMI	rs12401738	1	-0.0017	0.0041	$5e^{-8}$	A	G	171997	Alzheimer's disease and Dementia
High BMI	rs11165643	1	-0.0017	0.0042	$5e^{-8}$	T	C	171997	Alzheimer's disease and Dementia
High BMI	rs7531118	1	0.0006	0.0114	$5e^{-8}$	C	T	171997	Alzheimer's disease and Dementia
High BMI	rs3127553	1	0.00007	0.0008	$5e^{-8}$	A	G	171997	Alzheimer's disease and Dementia
High BMI	rs17024393	1	-0.0003	0.004	$5e^{-8}$	C	T	171997	Alzheimer's disease and Dementia
High BMI	rs12042908	2	-0.0006	0.0052	$5e^{-8}$	G	A	171997	Alzheimer's disease and Dementia
High BMI	rs6548237	2	0.0002	0.0039	$5e^{-8}$	C	A	171997	Alzheimer's disease and Dementia
High BMI	rs10182181	2	-0.002	0.0044	$5e^{-8}$	G	A	171997	Alzheimer's disease and Dementia
High BMI	rs1317006	2	-0.0010	0.0044	$5e^{-8}$	C	A	171997	Alzheimer's disease and Dementia
High BMI	rs1016287	2	-0.0002	0.0040	$5e^{-8}$	C	T	171997	Alzheimer's disease and Dementia

High BMI	rs13011109	2	0.063	0.0044	$5e^{-8}$	C	G	171997	Alzheimer's disease and Dementia
High BMI	rs12996547	2	0.0256	0.0049	$5e^{-8}$	T	C	171997	Alzheimer's disease and Dementia
High BMI	rs13417156	2	0.230	0.0096	$5e^{-8}$	C	T	171997	Alzheimer's disease and Dementia
High BMI	rs13098327	3	0.0341	0.0059	$5e^{-8}$	A	G	171997	Alzheimer's disease and Dementia
High BMI	rs16851483	3	-0.0259	0.0041	$5e^{-8}$	T	G	171997	Alzheimer's disease and Dementia
High BMI	rs1516725	3	0.0713	0.0040	$5e^{-8}$	C	T	171997	Alzheimer's disease and Dementia
High BMI	rs10938397	4	-0.0272	0.0057	$5e^{-8}$	G	A	171997	Alzheimer's disease and Dementia
High BMI	rs2112347	5	0.0687	0.0045	$5e^{-8}$	G	T	171997	Alzheimer's disease and Dementia
High BMI	rs9462027	6	0.0366	0.0051	$5e^{-8}$	A	G	171997	Alzheimer's disease and Dementia
High BMI	rs4615388	6	0.0244	0.0045	$5e^{-8}$	A	T	171997	Alzheimer's disease and Dementia
High BMI	rs2817419	6	-0.0254	0.0040	$5e^{-8}$	A	T	171997	Alzheimer's disease and Dementia
High BMI	rs12529728	6	0.0254	0.0043	$5e^{-8}$	G	A	171997	Alzheimer's disease and Dementia
High BMI	rs6465468	7	0.036	0.0039	$5e^{-8}$	T	C	171997	Alzheimer's disease and Dementia
High BMI	rs2060604	8	-0.0224	0.0041	$5e^{-8}$	C	T	171997	Alzheimer's disease and Dementia

High BMI	rs10968576	9	0.0229	0.0040	$5e^{-8}$	C	T	171997	Alzheimer's disease and Dementia
High BMI	rs1928295	9	0.0330	0.0050	$5e^{-8}$	G	A	171997	Alzheimer's disease and Dementia
High BMI	rs10733682	9	0.235	0.0059	$5e^{-8}$	C	T	171997	Alzheimer's disease and Dementia
High BMI	rs4929923	11	0.265	0.0044	$5e^{-8}$	G	A	171997	Alzheimer's disease and Dementia
High BMI	rs3817334	11	0.0310	0.0041	$5e^{-8}$	C	T	171997	Alzheimer's disease and Dementia
High BMI	rs11030107	11	0.0265	0.0042	$5e^{-8}$	T	C	171997	Alzheimer's disease and Dementia
High BMI	rs10767664	11	0.032	0.0039	$5e^{-8}$	G	A	171997	Alzheimer's disease and Dementia
High BMI	rs7138803	12	0.0348	0.0041	$5e^{-8}$	A	T	171997	Alzheimer's disease and Dementia
High BMI	rs4981693	14	0.0363	0.0059	$5e^{-8}$	A	G	171997	Alzheimer's disease and Dementia
High BMI	rs7141420	14	0.026	0.0058	$5e^{-8}$	T	C	171997	Alzheimer's disease and Dementia
High BMI	rs745213	15	0.0343	0.0044	$5e^{-8}$	G	T	171997	Alzheimer's disease and Dementia
High BMI	rs11074446	16	-0.0312	0.0060	$5e^{-8}$	C	T	171997	Alzheimer's disease and Dementia
High BMI	rs8055138	16	0.0262	0.0060	$5e^{-8}$	T	C	171997	Alzheimer's disease and Dementia
High BMI	rs7203521	16	0.0744	0.0042	$5e^{-8}$	A	G	171997	Alzheimer's disease and Dementia

High BMI	rs1121980	18	-0.0368	0.0054	$5e^{-8}$	A	G	171997	Alzheimer's disease and Dementia
High BMI	rs12446632	18	-0.0358	0.0043	$5e^{-8}$	A	G	171997	Alzheimer's disease and Dementia
High BMI	rs9956279	18	-0.0270	0.0023	$5e^{-8}$	T	C	171997	Alzheimer's disease and Dementia
High BMI	rs9954571	18	-0.0336	0.0039	$5e^{-8}$	A	G	171997	Alzheimer's disease and Dementia
High BMI	rs1563602	18	-0.0256	0.0027	$5e^{-8}$	C	T	171997	Alzheimer's disease and Dementia
High BMI	rs11663558	18	0.0567	0.0035	$5e^{-8}$	A	G	171997	Alzheimer's disease and Dementia
High BMI	rs663129	18	0.036	0.0056	$5e^{-8}$	A	G	171997	Alzheimer's disease and Dementia
High BMI	rs8097783	18	-0.0512	0.0037	$5e^{-8}$	A	G	171997	Alzheimer's disease and Dementia
High BMI	rs7239883	18	-0.0231	0.0076	$5e^{-8}$	A	G	171997	Alzheimer's disease and Dementia
High BMI	rs17066842	18	0.0567	0.0107	$5e^{-8}$	A	G	171997	Alzheimer's disease and Dementia
High BMI	rs11672660	18	-0.0512	0.0050	$5e^{-8}$	T	C	171997	Alzheimer's disease and Dementia
High BMI	rs2303108	19	-0.0231	0.0036	$5e^{-8}$	C	T	171997	Alzheimer's disease and Dementia
High BMI	rs6091540	20	-0.0297	0.0041	$5e^{-8}$	T	C	171997	Alzheimer's disease and Dementia

HD	rs1047891	2	-0.0054	0.345	$5e^{-8}$	A	C	26757	Alzheimer's disease and Dementia
HD	rs4299376	2	-0.0052	0.315	$5e^{-8}$	T	G	26757	Alzheimer's disease and Dementia
HD	rs72807674	2	0.0052	0.536	$5e^{-8}$	C	A	26757	Alzheimer's disease and Dementia
HD	rs9349379	6	0.0044	0.079	$5e^{-8}$	G	A	26757	Alzheimer's disease and Dementia
HD	rs376563	6	0.0056	0.093	$5e^{-8}$	C	G	26757	Alzheimer's disease and Dementia
HD	rs9295128	6	0.0410	0.056	$5e^{-8}$	T	T	26757	Alzheimer's disease and Dementia
HD	rs74617384	6	0.0210	0.013	$5e^{-8}$	T	G	26757	Alzheimer's disease and Dementia
HD	rs682216	6	0.0576	0.0088	$5e^{-8}$	T	C	26757	Alzheimer's disease and Dementia
HD	rs2071475	6	0.0060	0.0098	$5e^{-8}$	A	G	26757	Alzheimer's disease and Dementia
HD	rs1333047	9	0.0063	0.045	$5e^{-8}$	T	A	26757	Alzheimer's disease and Dementia
HD	rs8043119	15	0.0019	0.056	$5e^{-8}$	A	G	26757	Alzheimer's disease and Dementia

Remark: β stands for the estimated coefficient of exposures (GDM, High BMI and HD respectively) by a moderate screening LD criterion $(r^2 = 0.05, kb = 1000)$. P-value stands for genome-wide significant threshold (P = $5e^{-8}$) for screening SNPs significantly associated with exposures. This supplementary data recorded the genetic information of exposures, including Chromosome, Position, Effect allele and other Effect allele.

Supplementary data 2 : Extracting IVs of exposures in maternal AD/dementia

Exposure	Chromosome	Position	β	Standard deviation	*P*-value	Effect allele	Other effect allele	Sample size	Outcome
GDM	rs10830963	10	0.0006	0.0008	0.436	G	C	2062	Alzheimer's disease and Dementia
GDM	rs61872784	11	-0.0011	0.0008	0.151	A	T	2062	Alzheimer's disease and Dementia
High BMI	rs9462027	6	-0.0018	0.0008	0.023	A	G	171997	Alzheimer's disease and Dementia
High BMI	rs12401738	1	-0.0017	0.0007	0.016	A	G	171997	Alzheimer's disease and Dementia
High BMI	rs1317006	2	-0.0017	0.0007	0.03	C	T	171997	Alzheimer's disease and Dementia
High BMI	rs7531118	1	0.0006	0.0007	0.384	C	T	171997	Alzheimer's disease and Dementia
High BMI	rs4929923	11	0.00007	0.0008	0.922	C	T	171997	Alzheimer's disease and Dementia
High BMI	rs7141420	14	-0.0003	0.0009	0.631	T	C	171997	Alzheimer's disease and Dementia
High BMI	rs2060604	8	-0.0006	0.0007	0.414	C	T	171997	Alzheimer's disease and Dementia
High BMI	rs9956279	18	0.0002	0.002	0.808	T	C	171997	Alzheimer's disease and Dementia
High BMI	rs13098327	3	-0.002	0.0008	0.059	A	G	171997	Alzheimer's disease and Dementia
High BMI	rs12042908	1	-0.0010	0.003	0.775	G	A	171997	Alzheimer's disease and Dementia
High BMI	rs543874	1	-0.0002	0.0006	0.250	T	C	171997	Alzheimer's disease and Dementia

Exposure	Chromosome	Position	*β*	Standard deviation	*P*-value	Effect allele	Other effect allele	Sample size	Outcome
High BMI	rs9954571	1	-0.0002	0.0007	0.777	A	G	171997	Alzheimer's disease and Dementia
High BMI	rs8097783	18	-0.0001	0.0011	0.918	A	G	171997	Alzheimer's disease and Dementia
High BMI	rs11074446	18	0.0020	0.0014	0.144	C	T	171997	Alzheimer's disease and Dementia
High BMI	rs7203521	16	0.0011	0.0011	0.950	A	A	171997	Alzheimer's disease and Dementia
High BMI	rs745213	16	-0.0001	0.0007	0.128	A	G	171997	Alzheimer's disease and Dementia
High BMI	rs2303108	15	0.0009	0.0009	0.917	G	T	171997	Alzheimer's disease and Dementia
High BMI	rs17024393	19	0.0020	0.0008	0.243	C	T	171997	Alzheimer's disease and Dementia
High BMI	rs6465468	1	0.0002	0.0022	0.366	C	T	171997	Alzheimer's disease and Dementia
High BMI	rs17066842	7	-0.0015	0.0008	0.749	T	G	171997	Alzheimer's disease and Dementia
High BMI	rs11663558	18	0.0010	0.0018	0.413	A	G	171997	Alzheimer's disease and Dementia
High BMI	rs7138803	18	-0.0004	0.0007	0.147	A	G	171997	Alzheimer's disease and Dementia
High BMI	rs11672660	12	-0.0003	0.0007	0.599	A	G	171997	Alzheimer's disease and Dementia
High BMI	rs6091540	19	-0.0002	0.0009	0.722	T	C	171997	Alzheimer's disease and Dementia

High BMI	rs6091540	20	-0.0008	0.0008	0.827	T	C	171997	Alzheimer's disease and Dementia
High BMI	rs1928295	9	-0.0005	0.0007	0.280	C	T	171997	Alzheimer's disease and Dementia
High BMI	rs4981693	14	-0.0007	0.0008	0.558	A	G	171997	Alzheimer's disease and Dementia
High BMI	rs4615388	6	0.0009	0.0007	0.394	A	T	171997	Alzheimer's disease and Dementia
High BMI	rs1516725	3	-0.0020	0.0008	0.404	C	T	171997	Alzheimer's disease and Dementia
High BMI	rs12529728	6	-0.0001	0.0007	0.032	G	A	171997	Alzheimer's disease and Dementia
High BMI	rs10182181	2	-0.0009	0.0008	0.840	G	A	171997	Alzheimer's disease and Dementia
High BMI	rs1016287	2	0.0008	0.0008	0.257	C	T	171997	Alzheimer's disease and Dementia
High BMI	rs2112347	5	0.0014	0.0010	0.286	G	T	171997	Alzheimer's disease and Dementia
High BMI	rs3127553	1	0.0006	0.0009	0.054	A	G	171997	Alzheimer's disease and Dementia
High BMI	rs12446632	16	-0.0004	0.0008	0.523	A	G	171997	Alzheimer's disease and Dementia
High BMI	rs8055138	16	0.000	0.0007	0.078	C	C	171997	Alzheimer's disease and Dementia
High BMI	rs1121980	16	-0.0002	0.0008	0.061	G	G	171997	Alzheimer's disease and Dementia
High BMI	rs7239883	18	-0.0013	0.0008	0.796	G	G	171997	Alzheimer's disease and Dementia

High BMI	rs663129	18	0.0013	0.0004	0.523	A	G	171997	Alzheimer's disease and Dementia
High BMI	rs10733682	9	0.0002	0.0003	0.116	G	A	171997	Alzheimer's disease and Dementia
High BMI	rs11030107	11	0.0004	0.0002	0.490	G	A	171997	Alzheimer's disease and Dementia
High BMI	rs3817334	11	-0.0006	0.0003	0.611	T	C	171997	Alzheimer's disease and Dementia
High BMI	rs10767664	11	-0.0004	0.0002	0.412	A	T	171997	Alzheimer's disease and Dementia
High BMI	rs1563602	18	0.0000	0.0005	0.817	C	T	171997	Alzheimer's disease and Dementia
High BMI	rs13011109	2	0.0013	0.0013	0.128	C	G	171997	Alzheimer's disease and Dementia
High BMI	rs13417156	2	0.0002	0.0031	0.866	C	T	171997	Alzheimer's disease and Dementia
High BMI	rs6548237	2	0.0006	0.0032	0.671	C	A	171997	Alzheimer's disease and Dementia
High BMI	rs10968576	9	-0.0004	0.0006	0.156	G	A	171997	Alzheimer's disease and Dementia
High BMI	rs10938397	4	0.0000	0.0007	0.049	G	A	171997	Alzheimer's disease and Dementia
High BMI	rs12996547	2	-0.0002	0.0002	0.267	T	C	171997	Alzheimer's disease and Dementia
High BMI	rs16851483	3	-0.0018	0.0016	0.543	T	G	171997	Alzheimer's disease and Dementia
High BMI	rs2817419	6	0.0006	0.0008	0.388	A	G	171997	Alzheimer's disease and Dementia

HD	rs2071475	6	0.0003	0.0009	0.728	A	G	26757	Alzheimer's disease and Dementia
HD	rs1047891	2	-0.0007	0.0008	0.356	A	C	26757	Alzheimer's disease and Dementia
HD	rs1333047	9	0.0000	0.0007	0.985	T	A	26757	Alzheimer's disease and Dementia
HD	rs4299376	2	0.0015	0.0008	0.040	T	G	26757	Alzheimer's disease and Dementia
HD	rs682216	6	0.0001	0.0007	0.898	T	C	26757	Alzheimer's disease and Dementia
HD	rs9295128	6	-0.0020	0.0028	0.480	T	G	26757	Alzheimer's disease and Dementia
HD	rs72807674	2	0.0010	0.0008	0.903	C	A	26757	Alzheimer's disease and Dementia
HD	rs8043119	15	0.0003	0.0007	0.156	A	G	26757	Alzheimer's disease and Dementia
HD	rs376563	6	0.0003	0.0007	0.719	C	T	26757	Alzheimer's disease and Dementia
HD	rs9349379	6	0.0010	0.0007	0.692	G	A	26757	Alzheimer's disease and Dementia
HD	rs74617384	6	-0.0015	0.0013	0.239	T	A	26757	Alzheimer's disease and Dementia

Remark: β stands for the estimated coefficient of exposures (GDM, High BMI and HD) via extracting their SNPs in maternal AD/dementia genetic information after screening LD. This supplementary data recorded the genetic information of exposures, including Chromosome, Position, Effect allele and other Effect allele.

Supplementary Data 3 : Sensitivity analyses of the effects of exposure factors on maternal AD/dementia

Part 1: The results of heterogeneity test between GDM, high BMI, HD and maternal AD/dementia.

Table 2: The result of heterogeneity test between GDM and maternal Alzheimer's disease/Dementia.

Exposure	Method	Q	Q_df	*P*-value	Outcome
GDM	Inverse-variance weighted	3.04	1	0.11	Alzheimer's disease and Dementia

Remark: Q stands for the Q statistics from the Inverse-variance weighted method, and Q_df stands for the degree of freedom.

Table 3: The result of heterogeneity test between high BMI and maternal Alzheimer's disease/dementia.

Exposure	Method	Q	Q_df	*P*-value	Outcome
High BMI	Inverse-variance weighted	70.76	51	0.034	Alzheimer's disease and Dementia
High BMI	MR-Egger intercept	70.87	50	0.028	Alzheimer's disease and Dementia

Remark: Q stands for the Q statistics from the inverse-variance weighted method and MR-Egger intercept test. Q_df stands for the degree of freedom.

Table 4: The result of heterogeneity correction between high BMI and maternal Alzheimer's disease/Dementia.

Exposure	Method	N(SNPs)	β	Standard deviation	*P*-value	Outcome
High BMI	Multiple random-effects modeling	52	0.000068	0.0032	0.98	Alzheimer's disease and Dementia

Remark: N (SNPs) stands for the number of SNPs in high BMI. β stands for the estimated coefficient calculated by Multiple random-effects modeling method.

Table 5: The result of heterogeneity test between high BMI and maternal Alzheimer's disease/Dementia.

Exposure	MR-Egger intercept	Standard deviation	*P*-value	Outcome
High BMI	-0.000092	0.00033	0.77	Alzheimer's disease and Dementia

Remark: MR-Egger intercept stands for the intercept term calculated by the MR-Egger intercept test.

Table 6: The result of heterogeneity test between HD and maternal Alzheimer's disease/Dementia.

Exposure	Method	Q	Q_df	*P*-value	Outcome
HD	Inverse-variance weighted	13.11	9	0.15	Alzheimer's disease and Dementia
HD	MR-Egger intercept	10.20	8	0.25	Alzheimer's disease and Dementia

Remark: Q stands for the Q statistics from the inverse-variance weighted method and MR-Egger intercept test, and Q_df stands for the degree of freedom in Q statistics .

Table 7: The result of pleiotropy test between HD and maternal Alzheimer's disease/Dementia.

Exposure	MR-Egger intercept	Standard deviation	*P*-value	Outcome
HD	0.00066	0.00043	0.17	Alzheimer's disease and Dementia

Remark: MR-Egger intercept stands for the intercept term calculated by the MR-Egger intercept test.

Part 2 : Results of MVMR analysis and MR-PRESSO sensitivity test regarding the totally causal relationship between GDM, high BMI, HD and maternal Alzheimer's disease and dementia.

Table 9: The result of MR-PRESSO sensitivity test.

Method	RSS	*P*-value
MR-PRESSO Global Test	82.32	0.004

Remark: RSS stands for the residual sum of squares calculated by the MR-PRESSO Global Test.

Table 10: The result of MR-PRESSO outlier test.

Exposures	Causal estimation	Standard deviation	*P*-value
E1_effect	-0.0013	0.0016	0.43
E2_effect	-0.0013	0.0042	0.53
E3_effect	-0.078	0.046	0.10
E1_effect Outlier-corrected	NA	NA	NA
E1_effect Outlier-corrected	NA	NA	NA
E1_effect Outlier-corrected	NA	NA	NA

Remark: E1_effect stands for the effect of GDM exposure. E2_effect stands for the effect of high BMI exposure. E3_effect stands for the effect of HD exposure. Causal estimation means the estimated coefficient from the MR-PRESSO outlier test. If *P* value > 0.05, means non-significant sensitivity from the outlier test, then the result of Outlier-corrected displays “NA” in R software.

Table 11: The result of MR-PRESSO distortion test.

Method	Coefficient	Result
MR-PRESSO Distortion Test	No significant outliers	No significant outliers

Coefficient means the estimated coefficient from the MR-PRESSO Distortion Test, if there is no significant outlier before and after eliminating outliers, the “Coefficient” will display “No significant outliers” in R software, and the result of MR-PRESSO Distortion Test will display “No significant Outliers” in R software.

Supplementary data 4: The effect of exposures on outcome in MVMR analysis.

E1_Effect	E2_Effect	E3_Effect	E1_SE	E2_SE	E3_SE	Y_Effect	Y_SE
-0.0368	-0.0254	0.0005	0.0370	0.0044	0.0010	-0.0009	0.0007
-0.0009	0.0366	0.0007	0.0333	0.0039	0.0009	-0.0001	0.0006
0.0645	0.0176	-0.0054	0.0352	0.0046	0.0009	-0.0007	0.0007
0.0733	-0.0456	-0.0020	0.0434	0.0054	0.0011	0.0013	0.0008
0.3621	0.0114	0.0007	0.0344	0.0047	0.0010	0.0002	0.0007
0.0004	-0.0404	-0.0002	0.0327	0.0041	0.0009	0.0006	0.0006
-0.0142	-0.0289	-0.0002	0.0338	0.0043	0.0009	-0.0001	0.0007
0.0022	0.0230	0.0017	0.0334	0.0040	0.0009	-0.0017	0.0006
0.0367	0.0774	0.0005	0.0335	0.0039	0.0009	-0.0002	0.0006
-0.0271	-0.0270	-0.0007	0.0329	0.0046	0.0009	0.0013	0.0006
-0.0125	-0.0432	0.0029	0.0495	0.0058	0.0012	0.0010	0.0009
0.0797	0.0462	0.0033	0.0407	0.0051	0.0012	0.0006	0.0008
0.0314	-0.0333	0.0014	0.0432	0.0049	0.0011	-0.0020	0.0008
-0.0608	0.0524	0.0019	0.0769	0.0096	0.0017	0.0017	0.0012
-0.0814	-0.0713	-0.0031	0.0724	0.0114	0.0027	0.0005	0.0019
0.0487	0.0258	0.0006	0.0327	0.0039	0.0009	-0.0020	0.0006
-0.0373	-0.0232	-0.0011	0.0336	0.0040	0.0009	0.0008	0.0006
0.0350	0.0043	-0.0060	0.0468	0.0057	0.0011	-0.0006	0.0008
-0.0032	0.0298	0.0017	0.0333	0.0041	0.0009	-0.0003	0.0006
0.0327	-0.0278	-0.0012	0.0343	0.0043	0.0010	-0.0008	0.0007
0.0075	-0.0259	0.0000	0.0336	0.0042	0.0009	-0.0009	0.0007
-0.0449	-0.0265	-0.0008	0.0350	0.0040	0.0009	0.0014	0.0007
0.0089	-0.0107	0.0052	0.0410	0.0048	0.0009	0.0002	0.0008
0.0394	-0.0235	-0.0001	0.0345	0.0041	0.0009	-0.0015	0.0007
-0.0088	-0.0336	-0.0012	0.0350	0.0054	0.0010	-0.0001	0.0008
0.0494	0.0603	0.0020	0.0428	0.0050	0.0011	0.0005	0.0009
-0.0257	-0.0297	-0.0014	0.0390	0.0044	0.0010	-0.0010	0.0008
0.3112	-0.0183	0.0019	0.0433	0.0044	0.0010	-0.0002	0.0008
-0.0384	-0.0245	-0.0001	0.0384	0.0045	0.0009	-0.0011	0.0008
0.1182	0.0687	0.0003	0.0445	0.0052	0.0011	-0.0002	0.0009
0.0854	0.0567	-0.0003	0.0422	0.0046	0.0010	-0.0018	0.0008
0.0534	0.0348	0.0000	0.0338	0.0041	0.0009	0.0006	0.0007
0.0088	0.0231	0.0020	0.0331	0.0041	0.0009	-0.0004	0.0007
0.0271	0.0343	0.0013	0.0490	0.0053	0.0011	-0.0002	0.0009
0.1084	0.0244	-0.0210	0.0787	0.0125	0.0016	-0.0001	0.0013
0.0030	-0.0049	-0.0052	0.0350	0.0050	0.0009	0.0015	0.0007
0.1227	0.0512	0.0012	0.0824	0.0076	0.0017	0.0010	0.0014
0.0176	-0.0258	0.0015	0.0336	0.0042	0.0009	0.0020	0.0007

Remark: E1_Effect and E1_SE stand for the effect and the standard deviation of GDM calculated by Multivariable Inverse-variance weighted method. E2_Effect and E2_SE stand for the effect and the standard deviation of High BMI exposure calculated by Multivariable Inverse-variance weighted method. E3_Effect and E3_SE stand for the effect and the standard deviation of HD exposure calculated by Multivariable Inverse-variance weighted method. Y_Effect and Y_SE stand for the effect and the standard deviation of maternal AD/dementia outcome calculated by Multivariable Inverse-variance weighted method.
